# Supplementary material for: Exaiptasia pallida Infection Model Reveals the Critical Role of Vibrio parahaemolyticus T3SS Virulence Factors in Its Pathogenicity for Sea Anemones
Source: Toxins (Basel). 2025 Apr 2;17(4):175. doi: 10.3390/toxins17040175 (PMC12031060; doi:10.3390/toxins17040175)
Supplement: Supplementary file 1 [file toxins-17-00175-s001.zip › toxins-3531759-supplementary.pptx]

## Slide 1
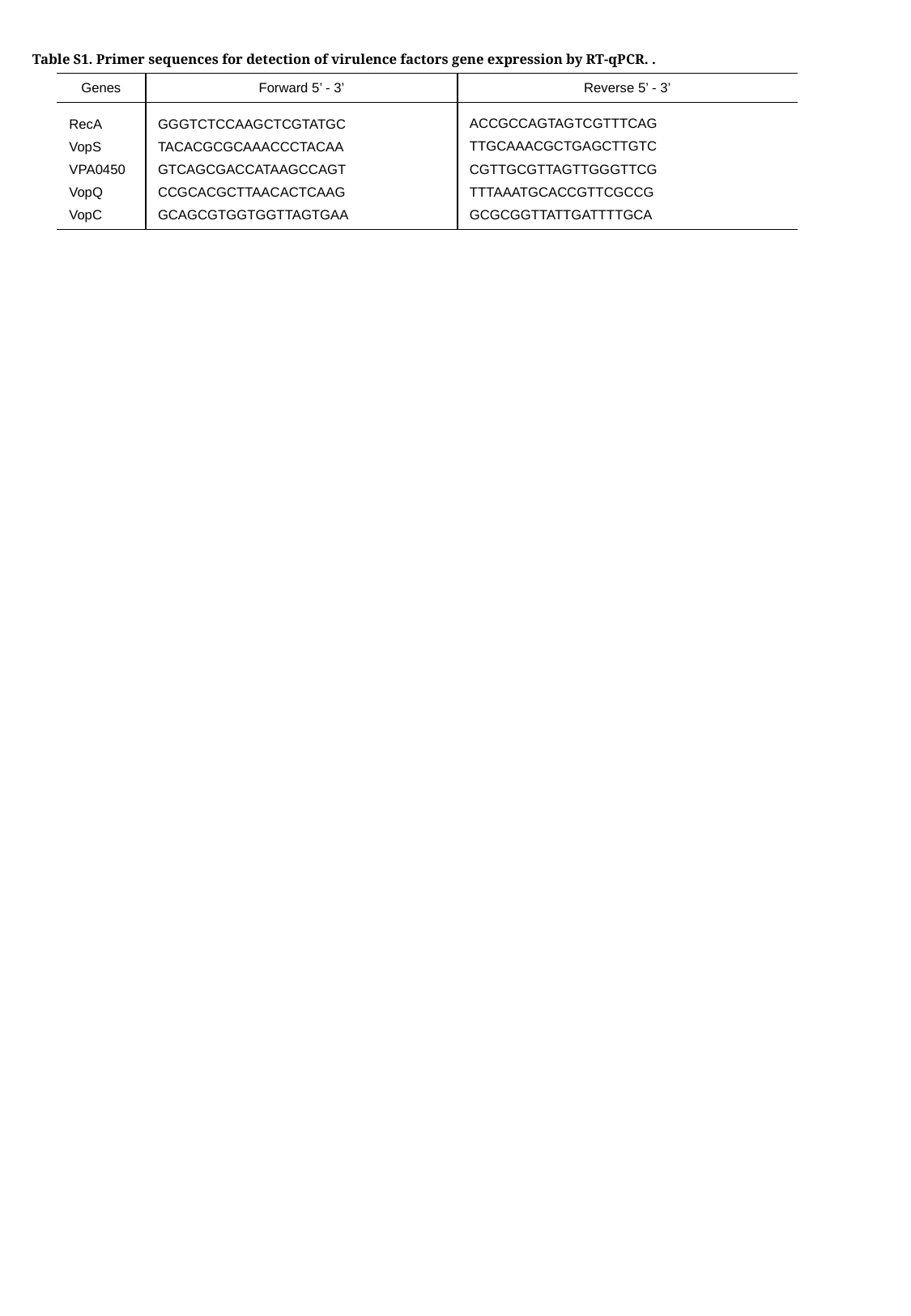

Table S1. Primer sequences for detection of virulence factors gene expression by RT-qPCR. .
| Genes | Forward 5’ - 3’ | Reverse 5’ - 3’ |
| --- | --- | --- |
| RecA VopS VPA0450 VopQ VopC | GGGTCTCCAAGCTCGTATGC TACACGCGCAAACCCTACAA GTCAGCGACCATAAGCCAGT CCGCACGCTTAACACTCAAG GCAGCGTGGTGGTTAGTGAA | ACCGCCAGTAGTCGTTTCAG TTGCAAACGCTGAGCTTGTC CGTTGCGTTAGTTGGGTTCG TTTAAATGCACCGTTCGCCG GCGCGGTTATTGATTTTGCA |

## Slide 2
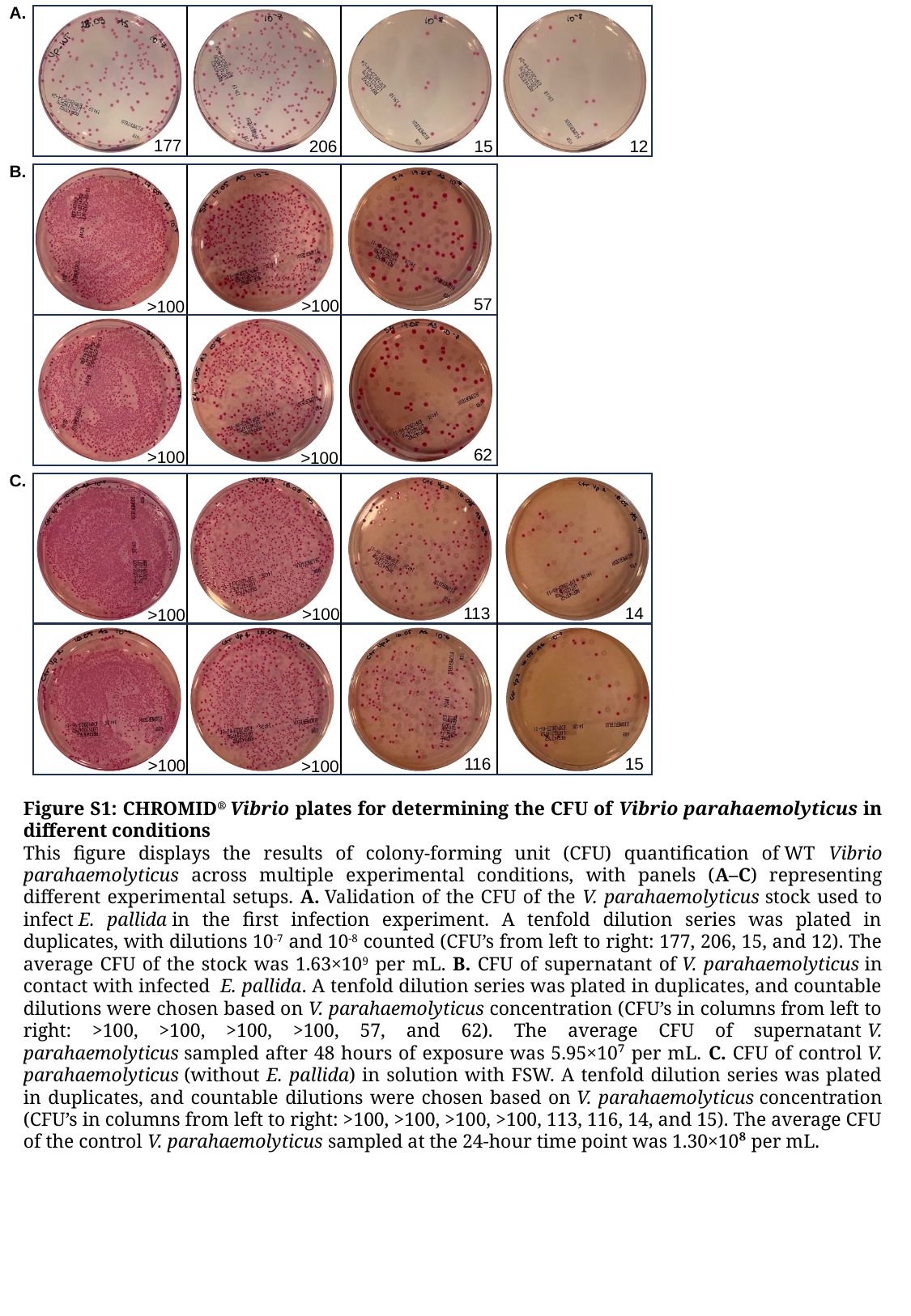

A.
177
12
206
15
B.
57
>100
>100
62
>100
>100
C.
113
14
>100
>100
116
15
>100
>100
Figure S1: CHROMID® Vibrio plates for determining the CFU of Vibrio parahaemolyticus in different conditions
This figure displays the results of colony-forming unit (CFU) quantification of WT Vibrio parahaemolyticus across multiple experimental conditions, with panels (A–C) representing different experimental setups. A. Validation of the CFU of the V. parahaemolyticus stock used to infect E. pallida in the first infection experiment. A tenfold dilution series was plated in duplicates, with dilutions 10-7 and 10-8 counted (CFU’s from left to right: 177, 206, 15, and 12). The average CFU of the stock was 1.63×10^9^ per mL. B. CFU of supernatant of V. parahaemolyticus in contact with infected  E. pallida. A tenfold dilution series was plated in duplicates, and countable dilutions were chosen based on V. parahaemolyticus concentration (CFU’s in columns from left to right: >100, >100, >100, >100, 57, and 62). The average CFU of supernatant V. parahaemolyticus sampled after 48 hours of exposure was 5.95×10⁷ per mL. C. CFU of control V. parahaemolyticus (without E. pallida) in solution with FSW. A tenfold dilution series was plated in duplicates, and countable dilutions were chosen based on V. parahaemolyticus concentration (CFU’s in columns from left to right: >100, >100, >100, >100, 113, 116, 14, and 15). The average CFU of the control V. parahaemolyticus sampled at the 24-hour time point was 1.30×10⁸ per mL.
